# Network Pharmacology-Based Approach to Investigate the Analgesic Efficacy and Molecular Targets of Xuangui Dropping Pill for Treating Primary Dysmenorrhea

**DOI:** 10.1155/2017/7525179

**Published:** 2017-10-08

**Authors:** Jihan Huang, Lei Li, Fan Cheung, Ning Wang, Yunfei Li, Zhenyu Fan, Fang Yin, Juan Yang, Rui Gao, Yingchun He, Yibin Feng

**Affiliations:** ^1^Center for Drug Clinical Research, Shanghai University of Traditional Chinese Medicine, Shanghai 201203, China; ^2^School of Chinese Medicine, Li Ka Shing Faculty of Medicine, The University of Hong Kong, Pokfulam, Hong Kong; ^3^Xiyuan Hospital of China Academy of Chinese Medical Sciences, Beijing 100091, China

## Abstract

This study aimed to evaluate the clinical analgesic efficacy and identify the molecular targets of XGDP for treating primary dysmenorrhea (PD) by a network pharmacology approach. Analysis of pain disappearance rate of XGDP in PD treatment was conducted based on data from phase II and III randomized, double-blind, double-simulation, and positive parallel controlled clinical trials. The bioactive compounds were obtained by the absorption, distribution, metabolism, and excretion processes with oral bioavailability (OB) and drug-likeness (DL) evaluation. Subsequently, target prediction, pathway identification, and network construction were employed to clarify the mechanisms of the analgesic effect of XGDP on PD. The pain disappearance rates in phase II and III clinical trials of XGDP in PD treatment were 62.5% and 55.8%, respectively, yielding a significant difference (*P* < 0.05) when compared with the control group using Tongjingbao granules (TJBG). Among 331 compounds, 53 compounds in XGDP were identified as the active compounds related to PD through OB, DL, and target prediction. The active compounds and molecular targets of XGDP were identified, and our study showed that XGDP may exert its therapeutic effects on PD through the regulation of the targets related to anti-inflammation analgesia and central analgesia and relieving smooth muscle contraction.

## 1. Introduction

Primary dysmenorrhea (PD) is a common gynecological complaint in adolescent girls and young women. It is characterized by lower abdominal pain and often accompanied by symptoms such as sweating, headache, nausea, vomiting, diarrhea, and tremulousness [1, 2]. Dysmenorrhea is common in adolescents, affecting up to 93% of the population [3]. Nonsteroid anti-inflammatory drugs (NSAIDs) are very effective for treating dysmenorrhea; however, NSAIDs are usually associated with many adverse effects including indigestion, headaches, and drowsiness [4].

Traditional Chinese Medicine (TCM) has been widely used in China to prevent, diagnose, and treat diseases over 2,000 years. Many Chinese medicines (CMs) have been used for treating PD and were proved to be effective without obvious side effects [5, 6]. Therefore, TCMs, including single herb and combination formulas, are perhaps the ideal therapeutics for treating PD [7]. Xuangui Dropping Pill (XGDP), which is a Chinese patent medicine, has been approved by China Food and Drug Administration (approval number Z20130008) and is widely used for treating dysmenorrhea in China.

XGDP is a modified classic TCM formula that is derived from the Xuangui Decoction in the Song dynasty Shenghui Prescription. Xuangui Decoction in TCM can activate blood circulation, dissipate stasis, and relieve pain and has been commonly used for the treatment of irregular menses and algomenorrhea. XGDP is composed of three Chinese medicinal herbs, namely, Corydalis Rhizoma (CR, Yanhusuo), Angelica Sinensis Radix (ASR, Danggui), and Zingiberis Rhizoma (ZR, Ganjiang). Among these three herbs, CR is a common herb that has been used as an analgesic in TCM for thousands of years [8], and modern pharmacological studies have shown that XGDP is a potential anti-inflammatory agent and analgesic [9, 10].

In recent years, much attention has been paid to TCM monomers and compounds. For example, the constituents in rat plasma after oral administration of CR were analyzed and identified using liquid chromatography-mass spectrometry/mass spectrometry [11]. However, the active substances and specific molecular mechanisms of the analgesic effect of XGDP for treating PD are still unclear.

A TCM formula is a complex combination with multiple components, multiple targets, and synergistic interactions among its components [12]. Because of the complex chemical composition, it is extremely difficult to study the role of the mixture system in the body. The complexity of TCM formulas makes it difficult to conduct a comprehensive study of TCM, whereas systems pharmacology [13] provides new ideas and perspectives for the study of Chinese herbal compounds. Identification of the active substances of TCM and their corresponding molecular targets and the determination of the relationship between active substances and diseases by using systems pharmacology [13] and network pharmacology [14] can help elucidate the molecular mechanisms of action of TCM formulas. For orally administered TCM formulas, the ingredients in the TCM formula must first overcome the barriers posed by the absorption, distribution, metabolism, and excretion (ADME) processes, and only the ingredients that can pass through the barriers may be active ingredients [15]. These ingredients can bind to the targets in the body and thus achieve efficacy by interacting with the human body at the network level and the overall organ level. The network pharmacology is applied to the study of TCM by combining oral bioavailability, drug-likeness screening, target identification, and network construction and analysis. The TCM network pharmacology not only offers an opportunity to discover bioactive ingredients and drug targets but also reveals the mechanisms of action between a TCM formula and the relevant diseases. For example, previous studies by the team of Yonghua Wang successfully applied TCM network pharmacology in the prediction of the active ingredients and potential targets [16], uncovering the molecular mechanism of medicinal herbs [17, 18] and the synergistic mechanisms [19].

**Figure 1:**
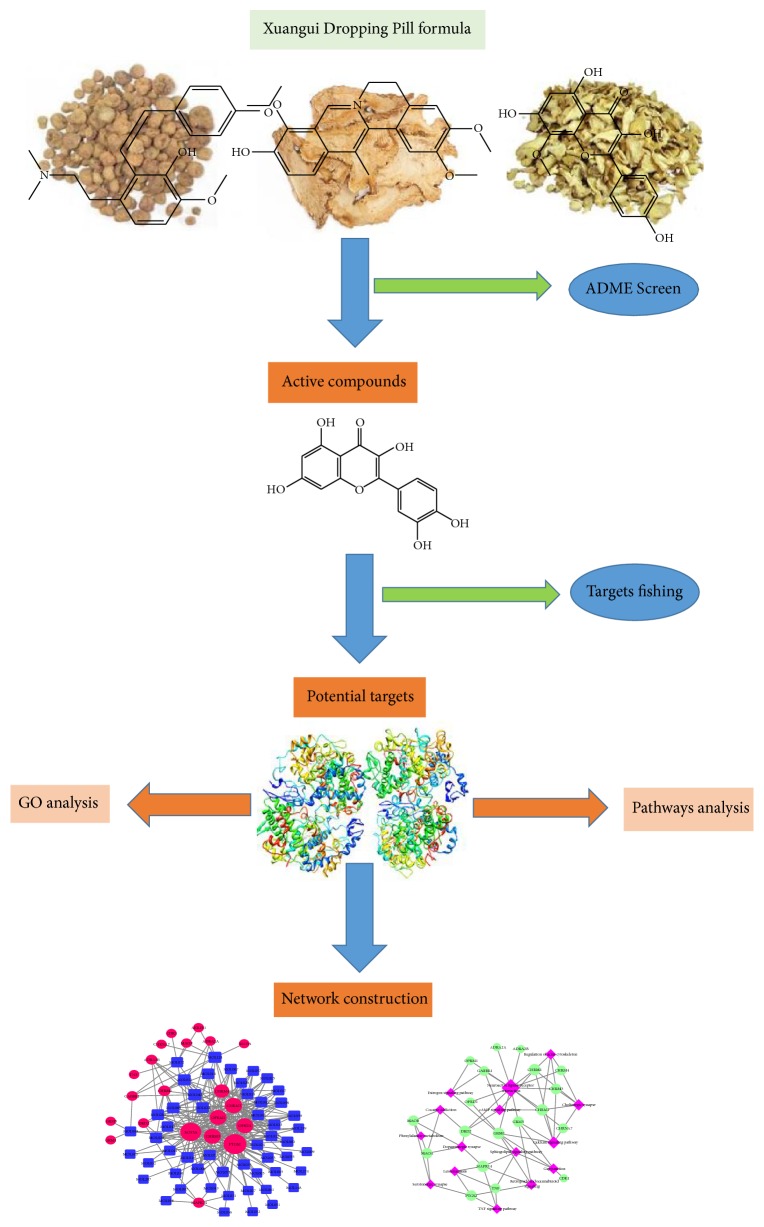
The workflow of the network pharmacological study of XGDP.

Therefore, in this study, we first evaluated the analgesic efficacy of XGDP in PD treatment based on data from phase II and III clinical trials. Then, on the basis of the ADME processes, we identified the active molecules of the XGDP formula that pass across the body barrier and predicted the network targets of the active substances. We also proposed its mechanisms of action on analgesia, which may provide a basis for an in-depth understanding of the mechanisms of action of XGDP for treating PD.  Figure 1 shows the workflow of network pharmacological study of XGDP.

## 2. Materials and Methods

### 2.1. Design of Clinical Trial

The clinical data in this study originated from phase II and III clinical trials before the approval of XGDP for sale. A randomized, double-blind, double-simulation, positive parallel controlled, and multicenter design was used in both clinical trials. Tongjingbao granules (TJBG) were used as a positive control drug. The TJBG has been approved by the China Food and Drug Administration (approval number Z41021972) and has analgesic and anti-inflammatory effects in treating dysmenorrhea [20]. The symptoms of the 240 and 480 patients with PD pain during the phase II and III clinical trials, respectively, were all consistent with the western medicine diagnosis of PD, as well as the TCM diagnosis of cold coagulation and blood stasis. The medicine sampled in this study was approved by the CFDA (approval number 2004L00160). The study protocol was approved by the ethics committee of the Affiliated Hospital of Hubei University of Chinese Medicine before study commencement. Each participant was informed of the purpose, processes, benefits, and risks of the study and signed a consent form.

### 2.2. Treatment Method

The treatment groups were given ten XGDP grains thrice per day; at the same time, they also took one packet of TJBG simulation agent thrice per day. The control group was given one packet of TJBG thrice per day; at the same time, they took ten grains of XGDP simulation agent thrice a day. The participants started their medication 3 days before their menstruation for 10 consecutive days. Three menstrual cycles constituted a single treatment.

### 2.3. Primary Outcome and Statistical Analysis

The primary outcome of the study was the pain disappearance rate. A full analysis set was used and all randomized participants were included in the analysis. A chi-square test was used to compare the differences between the two groups, with *P* ≤ 0.05 being the indicator of a statistically significant difference. The statistical analysis software used was SAS 9.3 (SAS Inst., Inc., Cary, NC, USA).

### 2.4. Candidate Compound Identification

Compound information of XGDP was retrieved from the Traditional Chinese Medicine Systems Pharmacology Database and Analysis Platform (TCMSP), which includes information of all 500 Chinese herbal medicines registered in the Chinese Pharmacopoeia (2010 edition) with a total of 30,069 ingredients collected through literature mining and database integration. Comprehensive data related to the pharmacokinetic properties of each chemical compound, comprising oral bioavailability (OB), intestinal epithelial permeability (Caco-2), drug likeness (DL), blood-brain barrier (BBB), drug half-life (HL), and Lipinski's rule (LR) of five, are provided for the screening and evaluation of the compounds [15].

### 2.5. Active Compound Screening

OB is one of the most crucial pharmacokinetic parameters in the ADME processes [21]. High OB is often used as the key indicator to determine the drug-like property of bioactive molecules. Most of the ingredients in TCMs fail to reach the protein target sites of particular cells because of the lack of appropriate pharmacological properties, particularly OB. In the present study, the molecules with OB ≥ 30% were considered to exhibit favorable OB.

In the early stages of drug development, the evaluation of DL helps to screen excellent compounds [16] and increases the hit rate of drug candidates. Therefore, in this study, the DL of the molecules in XGDP was assessed using the Tanimoto coefficient [22] through the following formula:

(1) $$\begin{array}{c} T\left(\;X,Y\;\right)=\frac{x*y}{x^{2}+y^{2}-x*y}, \end{array}$$

where *x* is the molecular descriptor of XGDP on the basis of Dragon software (TALETE) and *y* is the average descriptor of all drugs in the DrugBank database. Thus, in this study, the active molecules were defined as those with DL indices ≥0.15. The compounds with a DL index >0.15 were considered to exhibit more favorable DL and were selected as candidate molecules for further study.

### 2.6. Identification of Associated Proteins and Gene Names

The protein targets of the compounds were retrieved from the TCMSP database (http://lsp.nwu.edu.cn/tcmsp.php). The dataset used for building these models included 6511 drug molecules and 3987 targets with known compound–protein interactions in the DrugBank database [15]. The UniProt Knowledgebase (UniProtKB) is a protein database partially curated by experts and contains 54,247,468 sequence entries. Furthermore, the gene names were extracted from UniProtKB (http://www.uniprot.org). Finally, the targets were mapped to the Therapeutic Target Database (TTD, http://database.idrb.cqu.edu.cn/TTD/) to obtain the targets related to their corresponding diseases.

### 2.7. Identification of Significant Pathways

The Database for Annotation, Visualization, and Integrated Discovery (DAVID) bioinformatics resource comprises an integrated online biological knowledge base and analytical tools for systematically extracting biological data from large gene and protein lists (http://david.abcc.ncifcrf.gov/). In this study, to identify the pathways targeted by the compounds, the obtained genes were further analyzed using DAVID v6.7 [23]. Threshold count ≥2 and EASE scores ≤0.05 were selected in functional annotation clustering.

### 2.8. Network Construction and Analysis

The compound–target network was constructed using candidate compounds and potential targets. The target–pathway network was generated by linking the potential targets and the signaling pathways in which they participated. The networks were constructed using Cytoscape 3.3.0 software [24]. In this bilateral network, the nodes present the compounds, potential targets, or signal pathways, and the edges present the compound–target or target–pathway interactions.

## 3. Results

### 3.1. Demographic Characteristics

A total of 240 patients with PD were enrolled in the phase II clinical trial. 120 patients were randomized into the XGDP group; 116 of them completed the study and 4 of them dropped out. The other 120 patients were randomized into the TJBG group; 116 of them completed the study and 4 of them dropped out. The average ages of the XGDP and TJBG groups were 22.92 and 22.73 years, respectively; their respective average heights were 161.12 and 160.80 cm; their respective average weights were 51.04 and 50.58 kg; and their respective average courses of disease were 6.61 and 6.02 years (Table 1). There was no significant difference in demographic data between the two groups (*P* > 0.05).

Among the 480 patients with PD who were enrolled in the phase III clinical trial, 360 were randomized into the XGDP group, of whom 350 patients completed the study and 10 patients dropped out. A total of 120 patients were randomized into the TJBG group, with 116 of them completing the study and 4 of them dropping out. The average ages of the XGDP and TJBG groups were 22.55 and 22.95 years, respectively; their respective average heights were 161.42 and 160.80 cm; their respective average weights were 52.12 and 51.51 kg; and their respective average courses of disease were 5.03 and 5.69 years (Table 1). There was no significant difference in demographic data between the two groups (*P* > 0.05).

### 3.2. Pain Efficacy Analysis

**Figure 2:**
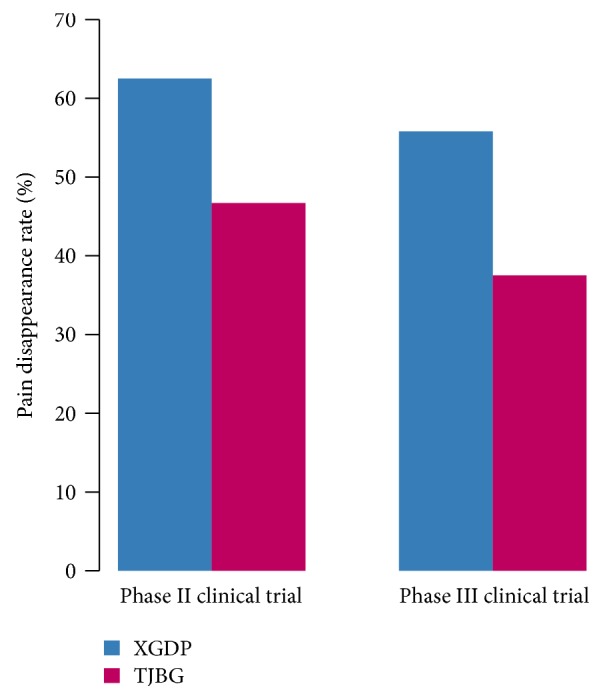
Analysis of pain disappearance rate of XGDP in PD pain treatment.

For 240 patients with PD who were enrolled in the phase II clinical trial, after three months of medication, 75 out of the 120 patients in the treatment group experienced abdominal pain relief with a 62.5% pain disappearance rate, compared with 56 out of the 120 patients in the control group with a 46.7% pain disappearance rate. The difference in the pain disappearance rate between the two groups was significant (*P* < 0.05). For 480 patients with PD who were enrolled in the phase III clinical trial with three months of medication, 201 out of the 360 patients in the treatment group experienced abdominal pain relief, achieving a 55.8% pain disappearance rate, compared with 45 out of the 120 patients in the control group, achieving a 37.5% pain disappearance rate (Figure 2). The difference in pain disappearance rate between the two groups was significant (*P* < 0.05).

### 3.3. Identification of Active Compounds of XGDP

A total of 350 compounds of XGDP were retrieved from the TCMSP database, comprising 77 in CR, 125 in ASR, and 148 in ZR. The three herbs shared the same 19 compounds (Supplementary 1, in the Supplementary Material available online at https://doi.org/10.1155/2017/7525179). Of the 77 compounds in CR, 54 satisfied the criteria of OB ≥ 30%, and 48 satisfied the criteria of OB ≥ 30% and DL ≥ 0.15. Of the 125 compounds in ASR, 69 satisfied the criteria of OB ≥ 30%, and 3 satisfied the criteria of OB ≥ 30% and DL ≥ 0.15. Of the 148 compounds in ZR, 58 satisfied the criteria of OB ≥ 30%, and 6 satisfied the criteria of OB ≥ 30% and DL ≥ 0.15. Among the 350 compounds, a total of 57 satisfied all aforementioned conditions (Table 2), and 55 compounds were obtained after removal of the duplicates. Many compounds of XGDP exhibited favorable OB, that is, 77.1%, 55.2%, and 39.1% of compounds in CR, ASR, and ZR, respectively. Moreover, 62.3% of compounds in CR exhibited favorable DL; however, a low percentage of compounds in ASR and ZR exhibited favorable DL.

The details of the 55 compounds are presented in Table 3. Notably, the medicinal herbs of CR and ASR both contained stigmasterol, and both ASR and ZR contained beta-sitosterol (Table 3).

For 55 compounds, most of the active compounds have been validated to display vital biological activities including analgesic and anti-inflammatory activities. For instance, tetrahydropalmatine (MOL016, OB = 73.94, DL = 0.64), corydaline (MOL022, OB = 65.84, DL = 0.68), and dehydrocorybulbine (DHCB) (MOL030, OB = 46.97, DL = 0.63) with favorable pharmacokinetic profiles come from CR, and they have a range of pharmacological properties including analgesic and anti-inflammatory activities [25–27]. Furthermore, stigmasterol (MOL124, OB = 43.83, DL = 0.76) and beta-sitosterol (MOL303, OB = 36.91, DL = 0.75), a main dietary phytosterol, may have anti-inflammatory effects via interfering with prostaglandin metabolism [28]. 6-Gingerol (MOL239, OB = 35.64, DL = 0.16) from ZR significantly inhibited the tumor necrosis factor-a (TNF-a) [29].

### 3.4. Identification of Targets of XGDP

**Figure 3:**
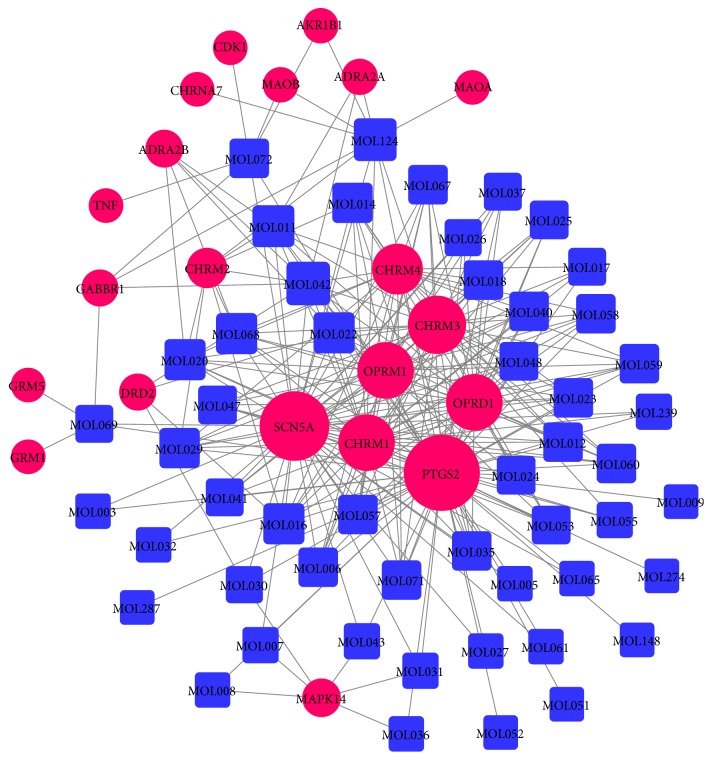
Compound–target network for XGDP for treating PD. The red nodes represent potential drug targets, and the blue nodes represent active compounds. The edges represent the interaction between them, and the node size is proportional to the degree.

For the 55 compounds, 1143 targets were obtained from the TCMSP (Supplementary 2), and 217 targets were finally obtained after removal of the duplicates. Subsequently, the targets were mapped to the TTD. Finally, 21 targets associated with dysmenorrhea were reserved (Table 4), and 53 compounds were finally obtained after removal of 2 candidate compounds (MOL303 and MOL304) without any relevant targets. The 53 candidate compounds and all their 21 potential targets were applied to generate a graph of compound–target interactions including 73 nodes (53 compounds and 21 targets) and 271 edges (Figure 3). The centralization and heterogeneity of the network were found to be 0.637 and 1.255, respectively. This finding indicates that some nodes are more concentrated in the network than others; that is, the compound–target space is biased toward certain compounds and targets. As shown in Figure 3, MOL042 (leonticine) displays the highest number of target interactions (degree = 12), followed by MOL124 (stigmasterol, degree = 11), MOL011 ((S)-scoulerine, degree = 10), MOL068 (isocorypalmine, degree = 9), MOL022 (corydaline, degree = 9), MOL020 (clarkeanidine, degree = 9), and MOL016 (tetrahydropalmatine, degree = 9). TCM is a multicomponent complex system; one component might act on multiple targets and act synergistically to treat diseases. In terms of target analysis, the targets showing a high degree may play a crucial role in the pharmacological function of XGDP. For instance, PTGS2 (degree = 52) was a major therapeutic target in inflammatory disease [30, 31]. SCN5A (degree = 44) was inhibited to be used for pain treatment which mediates the voltage-dependent sodium ion permeability of excitable membranes [32]. Both OPRD1 (delta-type opioid receptor) and OPRM1 (mu-type opioid receptor) corresponded to 28 compounds, which are involved in the regulation of central analgesic effects [33, 34]. Muscarinic acetylcholine receptors, CHRM3, CHRM1, and CHRM4, corresponded to 30, 28, and 21 compounds, respectively.

### 3.5. GO Biological Process Enrichment Analysis

**Figure 4:**
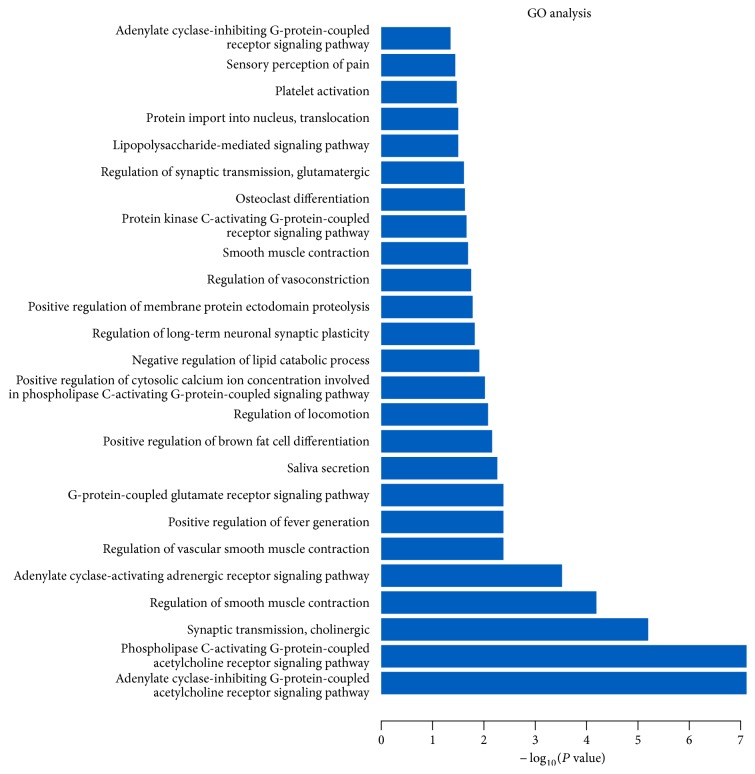
Gene analysis of treatment targets. The *x*-axis represents enrichment analysis ratings (*P* < 0.01) and the *y*-axis represents the enrichment analysis of significant types of biological processes.

The GO enrichment analysis function of the DAVID platform was used to study the predicted protein target function. A total of 25 entries were confirmed in this analysis (*P* < 0.05) in Figure 4. The major biological processes involved G-protein coupling that regulates smooth muscle contraction, transmits chemical synapses, and reacts to toxic substances. These findings demonstrated that the corresponding targets of XGDP and the corresponding genes of PD have functionally similar characteristics.

### 3.6. Identification of Significant Pathways

The Kyoto Encyclopedia of Genes and Genomes (KEGG) pathway enrichment analysis was performed using the functional annotation tool of DAVID Bioinformatics Resources 6.7. In total, 15 pathways were significantly associated with the input set of genes.

**Figure 5:**
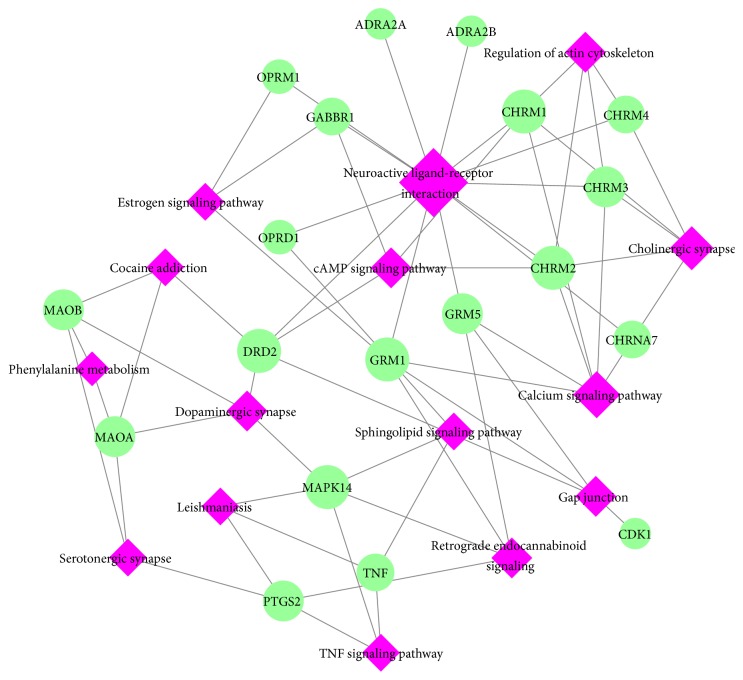
Target–pathway network of XGDP for treating PD. The pink nodes represent significant pathways, and the green nodes represent potential targets. The edges represent the interaction between them, and the node size is proportional to their degree.

To further elucidate the target–pathway mechanisms, a target–pathway network was constructed on the basis of all potential targets and their corresponding significant signaling pathways (Figure 5). As shown in Figure 5, this network is composed of 34 nodes (15 pathways and 19 proteins) and 64 interactions. The pink nodes represent the pathways, the green nodes represent the compounds, and the edges represent the interactions between them. The centralization and heterogeneity of the network were found to be 0.297 and 0.535, respectively. As shown in Figure 5, the results demonstrate that 13 nondisease pathways out of 15 pathways enriched within multiple targets of XGDP could be the key factors contributing to the anti-PD effect, such as neuroactive ligand-receptor interaction (degree = 13), calcium signaling pathway (degree = 6), cholinergic synapse (degree = 5), regulation of actin cytoskeleton (degree = 4), cAMP signaling pathway (degree = 4), dopaminergic synapse (degree = 4), retrograde endocannabinoid signaling (degree = 4), gap junction (degree = 4), sphingolipid signaling pathway (degree = 3), serotonergic synapse (degree = 3), TNF signaling pathway (degree = 3), and estrogen signaling pathway (degree = 3), and the other pathways interact with at least 2 potential targets. Neuroactive ligand-receptor interaction should be the crucial analgesic pathway and is regulated by 13 potential targets (OPRM1, DRD2, GABBR1, GRM1, GRM5, CHRM4, CHRM3, CHRM2, CHRM1, ADRA2A, CHRNA7, ADRA2B, and OPRD1). In addition, calcium signaling pathway possesses a wide range of cellular processes including contraction, exocytosis, and cell proliferation [35]. Calcium ions are important for cellular signaling and act in signal transduction resulting from the activation of ion channels or as a second messenger caused by indirect signal transduction pathways such as G-protein-coupled receptors. Nevertheless, cAMP is one of the most common and universal second messengers and cAMP signaling pathway regulates pivotal physiologic processes including calcium homeostasis, muscle contraction, metabolism, secretion, cell fate, and gene transcription [36]. Tumor necrosis factor (TNF), as a critical cytokine, can induce a wide range of intracellular signal pathways including inflammation and immunity [37]; the analgesic effects appeared to be mediated by inhibition of PTGS2 biosynthesis [25].

## 4. Discussion

A TCM formula is composed of multiple ingredients and has a complex mechanism of action, which may be associated with multiple targets and multiple pathways in humans. XGDP is a new drug commonly used for treating PD and has been approved by the China Food and Drug Administration. In the present study, a network pharmacology approach was applied to identify the bioactive compounds and significant pathways of XGDP by using OB and DL evaluation. In this study, 350 compounds of XGDP were extracted from the TCMSP database. The results showed that 55 compounds exhibited favorable OB and DL properties. Screening revealed that 53 compounds are the potential active molecules of XGDP, and these compounds will be selected as candidates for further study.

Among the 53 active compounds, a few compounds have been verified to possess multiple pharmacological actions including analgesic effects. Particularly, the tetrahydropalmatine (THP) exerted analgesic effects through blocking of voltage-activated L-type calcium channel active potassium channels on primary dysmenorrhea [25]. The enantiomer Levo-tetrahydropalmatine (Levo-THP) was synthetically produced by the pharmaceutical industry and has been marketed worldwide under different brand names as an alternative drug of analgesics. A recent study reported that dehydrocorybulbine (DHCB) is effective against inflammatory pain and injury-induced neuropathic pain and causes no antinociceptive tolerance interaction with D2 receptors [26, 38]. Eight isoquinoline alkaloids, that is, tetrahydropalmatine, corydaline, protopine, berberine, palmatine, jatrorrhizine, coptisine, and dehydrocorydaline, were isolated from the methanolic extract of the tubers of CR and they also were acetylcholinesterase inhibitors [39].

In the present study, through analysis of the topological properties of the compound–target interaction network, we found that compounds with a high degree and potential targets that occupy hub positions in the network may play crucial roles in the pharmacological function of XGDP. The analysis of the compound–target network showed that the main active ingredients of XGDP can act on multiple pathways, consistent with the multicomponent, multitarget, and integrated regulation characteristics of TCM formulas [12].

Dysmenorrhea may be related to nitric oxide (NO) [40] where XGDP can reduce NO levels through the calcium signaling pathway and arginine and proline metabolism, whereas the PTGS2 is the target for NSAIDs and PTGS2 (COX-2) specific inhibitors called coxibs for dysmenorrhea [41]. Notably, 52 compounds among XGDP act on PTGS2. The endogenous system of opioid receptors (OPRM1 and OPRD1) is well known for its analgesic potential; XGDP may treat dysmenorrhea through regulating central analgesic effects [33, 34]. The CHRM3 are located at many places including smooth muscles where they can cause smooth muscle contraction and increased glandular secretions. XGDP may relief smooth muscles shrinking by inhibiting CHRM3 receptor. It is interesting that many compounds exhibit analgesic effects in our study. Therefore, the multidirectional pharmacological treatment mechanisms of TCM formulas that act through multiple targets, anti-inflammation analgesia, central analgesia, and relieving smooth muscle contraction may be a main therapeutic strategy to treat PD. On the basis of the potential targets of the 53 compounds, 15 significant pathways associated with 19 potential targets were obtained. Our study showed that XGDP may exert its therapeutic effects on PD through neuroactive ligand-receptor interaction, calcium signaling pathway, cAMP signaling pathway, tumor necrosis factor (TNF) pathway, and so forth.

Phase II and III clinical trials of XGDP have verified its analgesic effect on patients with PD. This study further analyzed the possible chemical compositions that could enter the bloodstream using systems pharmacology and predicted the possible targets of the compositions through network pharmacology, after which GO and pathway enrichment analysis were conducted on the targets. The major active ingredients of XGDP can act on several pathways, displaying the characteristics of multicomponent, multitarget, and integrated regulation of TCM compounds. The complex composition of TCM that acts on distinct targets through systematic actions in all aspects of disease could achieve the purpose of disease treatment through mutual coordination.

The constituents of TCM are complex, and these ingredients with different effects and different targets can act on the various aspects of the disease through multiple systems, and they interact with each other to produce synergistic effects [42, 43]. Previous one-target and one-drug model studies have tended to ignore the relationship between many diseases [44]. A TCM formula is a multicomponent and multitarget synergistic system that accounts for this relationship. Network pharmacology can predict the target profiles and pharmacological actions of herbal compounds. In our study, network construction approaches were used to identify the bioactive compounds of XGDP and their potential targets and we preliminarily explored the analgesia mechanisms of XGDP for PD. For the complexity of TCM, potential targets of the 53 main compounds were further experimentally validated for their roles in relief of analgesia.

## 5. Conclusions

Among 331 compounds, a total of 53 bioactive compounds in XGDP with 15 significant pathways were identified through network analysis. Thus, the mechanism of action of XGDP for PD involves multiple compounds, targets, and pathways. The therapeutic effects of XGDP on PD may be dependent on the regulation of the targets related to anti-inflammation analgesia, central analgesia, and relieving smooth muscle contraction. The network pharmacology approaches developed in our study provide an alternative strategy for the comprehensive understanding of the therapeutic effects of XGDP on PD.

## Supplementary Material

Supplementary 1 displays the information of 350 compounds in Xuangui Dropping Pill. Supplementary 2 shows the information of the 55 compounds and 1143 targets of Xuangui Droping Pill.

## Figures and Tables

**Table 1:** Demographic characteristics.

Characteristics	XGDP group	TJBG group	*P* value
Phase II study	*N* = 120	*N* = 120	
Age (yr)	22.92 ± 3.04	22.73 ± 2.53	0.613
Height (cm)	161.12 ± 4.66	160.80 ± 4.60	0.597
Weight (kg)	51.04 ± 5.39	50.58 ± 5.97	0.526
Course of disease (yr)	6.61 ± 3.47	6.02 ± 3.19	0.170
Phase III study	*N* = 360	*N* = 120	
Age (yr)	22.55 ± 3.28	22.95 ± 3.40	0.249
Height (cm)	161.42 ± 4.61	160.80 ± 4.65	0.206
Weight (kg)	52.12 ± 5.73	51.51 ± 5.95	0.320
Course of disease (yr)	5.03 ± 3.24	5.69 ± 3.99	0.100

**Table 2:** Number of compounds in XGDP that satisfied OB ≥ 30% and DL ≥ 0.15.

Herbs	Total	OB ≥ 30%	DL ≥ 0.15
CR	77	54 (77.1)	48 (62.3)
ASR	125	69 (55.2)	3 (2.4)
ZR	148	58 (39.1)	6 (4.1)

**Table 3:** Information on candidate active compounds from CR, ASR, and ZR herbs.

Number	Molecule name	OB (%)	DL	PubChem CID	Herbs
MOL003	Berberine	36.86	0.78	2353	CR
MOL005	Coptisine	30.67	0.86	72322	CR
MOL006	Cryptopine	78.74	0.72	72616	CR
MOL007	Dihydrochelerythrine	32.73	0.81	485077	CR
MOL008	Dihydrosanguinarine	59.31	0.86	124069	CR
MOL009	Sanguinarine	37.81	0.86	5154	CR
MOL011	(S)-Scoulerine	32.28	0.54	439654	CR
MOL012	Cavidine	35.64	0.81	193148	CR
MOL014	(R)-Canadine	55.37	0.77	443422	CR
MOL016	Tetrahydropalmatine	73.94	0.64	72301	CR
MOL017^*∗*^	(−)-Alpha-N-methylcanadine	45.06	0.8	N/A	CR
MOL018^*∗*^	Capaurine	62.91	0.69	94149	CR
MOL020^*∗*^	Clarkeanidine	86.65	0.54	127376	CR
MOL022^*∗*^	Corydaline	65.84	0.68	101301	CR
MOL023^*∗*^	Corydalmine	52.5	0.59	161665	CR
MOL024^*∗*^	Corydine	37.16	0.55	10153	CR
MOL025^*∗*^	18797-79-0	46.06	0.85	177014	CR
MOL026^*∗*^	Corynoloxine	38.12	0.6	101324793	CR
MOL027^*∗*^	Methyl-[2-(3,4,6,7-tetramethoxy-1-phenanthryl)ethyl]amine	61.15	0.44	11462401	CR
MOL029^*∗*^	Dehydrocavidine	38.99	0.81	92043552	CR
MOL030^*∗*^	Dehydrocorybulbine	46.97	0.63	101879963	CR
MOL031^*∗*^	Dehydrocorydaline	41.98	0.68	34781	CR
MOL032^*∗*^	Dehydrocorydalmine	43.9	0.59	3083983	CR
MOL035^*∗*^	Demethylcorydalmatine	38.99	0.54	N/A	CR
MOL036^*∗*^	13-Methyldehydrocorydalmine	35.94	0.63	25254728	CR
MOL037^*∗*^	(1S,8′R)-6,7-Dimethoxy-2-methylspiro[3,4-dihydroisoquinoline-1,7′-6,8-dihydrocyclopenta[g][1,3]benzodioxole]-8′-ol	43.95	0.72	21770852	CR
MOL040	Izoteolin	39.53	0.51	133323	CR
MOL041^*∗*^	Isocorybulbine	40.18	0.66	12310873	CR
MOL042^*∗*^	Leonticine	45.79	0.26	12314123	CR
MOL043^*∗*^	13-Methylpalmatrubine	40.97	0.63	12275616	CR
MOL047^*∗*^	N-Methyllaurotetanine	41.62	0.56	6543699	CR
MOL048^*∗*^	Norglaucine	30.35	0.56	N/A	CR
MOL051^*∗*^	Pontevedrine	30.28	0.71	11047165	CR
MOL052^*∗*^	Pseudocoptisine	38.97	0.86	15520811	CR
MOL053^*∗*^	24240-05-9	53.75	0.83	185559	CR
MOL055^*∗*^	Saulatine	42.74	0.79	185141	CR
MOL057	Stylopine	48.25	0.85	440583	CR
MOL058^*∗*^	Tetrahydrocorysamine	34.17	0.86	14315597	CR
MOL059^*∗*^	Tetrahydroprotopapaverine	57.28	0.33	40512630	CR
MOL060^*∗*^	ST057701	31.87	0.56	6992288	CR
MOL061	2,3,9,10-Tetramethoxy-13-methyl-5,6-dihydroisoquinolino[2,1-b]isoquinolin-8-one	76.77	0.73	10362429	CR
MOL065	Palmatine	64.6	0.65	19009	CR
MOL067	Fumarine	59.26	0.83	4970	CR
MOL068	Isocorypalmine	35.77	0.59	10220	CR
MOL069	Bicuculline	69.67	0.88	10237	CR
MOL071	C09367	47.54	0.69	12441	CR
MOL072	Quercetin	46.43	0.28	5280343	CR
MOL124	Stigmasterol	43.83	0.76	5280794	ASR/CR
MOL148	2,6-Di(phenyl)thiopyran-4-thione	69.13	0.15	11832833	ASR
MOL236	1-Monolinolein	37.18	0.30	6436630	ZR
MOL239	6-Gingerol	35.64	0.16	442793	ZR
MOL274	[(1S)-3-[(E)-But-2-enyl]-2-methyl-4-oxo-1-cyclopent-2-enyl] (1R,3R)-3-[(E)-3-methoxy-2-methyl-3-oxoprop-1-enyl]-2,2-dimethylcyclopropane-1-carboxylate	62.52	0.31	5315890	ZR
MOL287	Sexangularetin	62.86	0.3	5281698	ZR
MOL303	Beta-sitosterol	36.91	0.75	222284	ZR/ASR
MOL304	Sitosterol	36.91	0.75	12303645	ZR

^*∗*^Compounds only present in CR.

**Table 4:** Information on 21 potential targets.

Number	Protein name	Gene name	UniProt ID
TAR01	Prostaglandin G/H synthase 2	PTGS2	P35354
TAR02	Sodium channel protein type 5 subunit alpha	SCN5A	Q14524
TAR03	Muscarinic acetylcholine receptor M3	CHRM3	P20309
TAR04	Muscarinic acetylcholine receptor M1	CHRM1	P11229
TAR05	Muscarinic acetylcholine receptor M4	CHRM4	P08173
TAR06	Delta-type opioid receptor	OPRD1	P41143
TAR07	Mu-type opioid receptor	OPRM1	P35372
TAR08	Mitogen-activated protein kinase 14	MAPK14	Q16539
TAR09	Alpha-2A adrenergic receptor	ADRA2A	P08913
TAR10	Muscarinic acetylcholine receptor M2	CHRM2	P08172
TAR11	Alpha-2B adrenergic receptor	ADRA2B	P18089
TAR12	D(2) dopamine receptor	DRD2	P14416
TAR13	Gamma-aminobutyric acid receptor subunit alpha-1	GABBR1	Q9UBS5
TAR14	Metabotropic glutamate receptor 5	GRM5	P41594
TAR15	Metabotropic glutamate receptor 1	GRM1	Q13255
TAR16	Aldose reductase	AKR1B1	P15121
TAR17	Amine oxidase [flavin-containing] B	MAOB	P27338
TAR18	Tumor necrosis factor	TNF	P01375
TAR19	Cell division control protein 2 homolog	CDK1	P06493
TAR20	Amine oxidase [flavin-containing] A	MAOA	P21397
TAR21	Neuronal acetylcholine receptor protein, alpha-7 chain	CHRNA7	P36544
